# The impact of COVID‐19 on nurse alcohol consumption: A qualitative exploration

**DOI:** 10.1111/jocn.16467

**Published:** 2022-07-24

**Authors:** Adam Searby, Dianna Burr, Bernice Redley

**Affiliations:** ^1^ Deakin University, Institute for Health Transformation School of Nursing & Midwifery Geelong Australia

**Keywords:** alcohol consumption, alcohol drinking, COVID‐19, nurses, nursing staff

## Abstract

**Aims and objectives:**

To explore the long‐term impact of the COVID‐19 pandemic on nurse alcohol consumption.

**Background:**

The COVID‐19 pandemic has caused immense disruption to healthcare services worldwide, and nurses have not been immune, experiencing burnout, declining mental health and ultimately, attrition from the profession. Increases in alcohol consumption have been reported across subsections of society, including those with pre‐existing mental ill health and experiencing high stress, and exploring this phenomenon in nurses is essential for workforce well‐being and sustainability.

**Design:**

Qualitative descriptive study design.

**Methods:**

Secondary analysis of individual, semi‐structured interviews with nurses (*N* = 42) from diverse settings across Australia, including community, primary and hospital settings, conducted in July and August 2021. Data were analysed using structural coding and reported in accordance with the CORE‐Q guidelines.

**Findings:**

Two key themes were found after analysis of the data: (1) factors influencing alcohol consumption (subthemes: workplace factors and external factors), and (2) the pandemic's influence on alcohol consumption (subthemes: increased consumption, moderation of consumption and alcohol as a reward).

**Conclusions:**

Several participants described increased alcohol consumption because of the COVID‐19 pandemic, particularly due to the stress of working in an environment where resources were scarce. Workplace factors such as overtime, missed breaks and heightened workload were all described as driving stress, and in turn increased alcohol consumption.

**Relevance to clinical practice:**

Increased alcohol consumption has been associated with burnout, absenteeism and intention to leave. The nursing profession is currently undergoing significant continuing stress providing care and management to patients with the SARS‐CoV‐2 virus, and increased alcohol consumption is a significant threat to personal and workforce well‐being, workforce sustainability and quality nursing care.

**What does this paper contribute to the wider clinical community?**

- Nurses in our study describe increasing alcohol consumption in response to staffing and resource shortages and workforce pressures occurring during the COVID‐19 pandemic.
- There is an urgent need to explore alcohol consumption among nurses to determine whether increases in consumption described during the pandemic continue, particularly regarding the ongoing impact on workforce sustainability and personal well‐being.

## 1 INTRODUCTION

The COVID‐19 pandemic has had a marked impact on society, including disruption to healthcare services and workers (Chew et al., 2020; Eftekhar Ardebili et al., 2021; Marvaldi et al., 2021). The SARS‐CoV‐2 virus is believed to have originated in 2019, causing extensive illness globally. The virus resulted in lockdowns, social distancing and isolation, travel restrictions and ‘work from home’ orders in 2020–2021 to reduce transmission and infection (Adekunle et al., 2020; Wiersinga et al., 2020). At the time of writing, the COVID‐19 pandemic is believed to have contributed to approximately 6.3 million deaths globally from a total of approximately 520 million infections (World Health Organization, 2022). Although Australia initially responded well to the challenge of COVID‐19, several waves of infection were experienced, resulting in several lockdowns and interstate border closures, only ceasing when vaccination rates had reached a level considered by public health experts to be protective to both society and the healthcare system (considered to be 90% of the adult population vaccinated), which was not prepared or resourced for a huge influx of COVID patients (Australian Institute of Health and Welfare, 2020; Hanly et al., 2022).

The COVID‐19 pandemic was attributed to increased alcohol consumption, particularly in the early phases of the pandemic with research conducted in the United States indicating that lockdowns and ‘stay at home’ orders were associated with both increasing drinking and deteriorating mental health among a number of individuals (Conroy Deirdre et al., 2021; Killgore et al., 2021). Frontline healthcare workers, portrayed throughout the pandemic as the face of healthcare services, have experienced increasing demand for healthcare services. Nurses have faced procedural changes that require higher numbers of resources (such as ventilation of COVID‐19 patients), visitor restrictions that have resulted in higher levels of aggression and violence from healthcare consumers, and the flow on effects of high healthcare demand that have resulted in burnout and occupational stress (Digby et al., 2021). Despite literature indicating high stress can result in increased alcohol use (Stanton et al., 2020), little research has explored alcohol consumption specifically among nurses during the COVID‐19 pandemic.

The aim of this paper is to explore the long‐term impact of the COVID‐19 pandemic on nurse alcohol consumption by exploring perceptions of the COVID‐19 pandemic and workplace stress. We sought to use a qualitative descriptive methodology to provide an in‐depth exploration of the perceptions of Australian nurses regarding the pandemic, workplace stress and possible impacts on their alcohol consumption.

### 1.1 Background

Research has found an emerging trend of long‐term impacts on the nursing workforce related to the demands of the COVID‐19 pandemic, including reports of burnout, declining mental health and the intention to leave the nursing profession entirely. For example, Holton et al. (2021) found 29% of nurses and midwives had anxiety scores in the mild to extremely severe range when administering the Depression, Anxiety and Stress Scale (DASS‐21) to 668 Australian health professionals. Intention to leave or quit the nursing profession has been reported in several studies internationally, including studies that have found an association between higher intention to leave in nurses who are caring for individuals with the SARS‐CoV‐2 virus (Lavoie‐Tremblay et al., 2022), and among nursing students (Nie et al., 2021).

Previous research exploring the mental health impact of the COVID‐19 pandemic on healthcare professionals has found reports of using alcohol to cope with heightened workplace stress. Foli et al.'s (2021) qualitative survey of 105 nurses in the United States of America found several reports of nurses turning to substances such as alcohol to cope. In this study, nurses also completed the Alcohol Use Disorders Identification Test (AUDIT) (Babor et al., 2001), recording a mean score of 4.3. The AUDIT is a highly validated instrument sensitive to several types of problematic alcohol consumption; however, under most AUDIT scoring matrices, this is considered a ‘low‐risk’ score, with scores over eight indicating high‐risk alcohol consumption (Babor et al., 2001).

In contrast, our recent cross‐sectional survey study of 1209 Australian nurses and midwives drawn from a wide variety of Australian clinical settings (Searby et al., 2022) found a mean AUDIT score of 7.11; higher scores were noted in specific settings likely to be impacted by the influx of COVID‐19 patients, such as the emergency department (mean 10.07), and the intensive care unit (mean 9.36). Further, our work also found a strong correlation between stress and alcohol consumption on a self‐reported perceptions of work stress instrument (the Perceived Work Stress Scale [Mackie et al., 2001]). These results were echoed in a study of 709 clinicians working in intensive care units in the United Kingdom, with nurses reporting poorer mental health than doctors participating in the survey (Greenberg et al., 2021).

Reported increases in alcohol consumption among healthcare workers are not consistent. For example, a study including healthcare workers (*n* = 381) in New Zealand did not find increased alcohol use, despite a higher rate of poor mental well‐being among healthcare workers (Bell et al., 2021). However, a cross‐sectional examination of healthcare workers in intensive care units (*n* = 342) reported increased alcohol consumption of 22.2% among participants and lower mental health scores (Wozniak et al., 2021). Consistently, increased alcohol consumption was correlated with lower scores on four measures of mental well‐being.

The longer‐term impact of the COVID‐19 pandemic on alcohol consumption is unknown; due to the rapid escalation of SARS‐CoV‐2 infections around the world, most research conducted has been cross‐sectional. However, longitudinal work conducted prior to the pandemic that considers stress and alcohol consumption has found a complex relationship between perceived work stress and increased drinking (Marchand & Blanc, 2011; Siegrist & Rödel, 2006). It should be noted, however, that previous studies have been conducted in the absence of a significant event such as a pandemic; the study most closely approximating these conditions was conducted in the aftermath of the September 11 terrorist attack in New York, finding that women experiencing chronic work stress were most likely to increase their alcohol consumption after this event (Richman et al., 2004).

### 1.2 Study aim

The aim of this study was to explore the long‐term impact of the COVID‐19 pandemic on nurse alcohol consumption by exploring their perceptions of the COVID‐19 pandemic and workplace stress.

## 2 METHODS

### 2.1 Design

We used qualitative description as the overarching methodology for this study. Qualitative description is defined as a naturalistic, ‘low inference’ method of qualitative research, where the description of events is provided in the participant's own language (Neergaard et al., 2009). Semi‐structured interviews were conducted with 42 nurses across Australia, and data analysed using structural coding (Saldaña, 2013). We used this approach as it is recommended where a study needs to address specific research aims or questions, in this case participant perceptions of the COVID‐19 pandemic on their alcohol consumption (Bradshaw et al., 2017). Ethical approval for this study was obtained from a local health service and endorsed by the relevant university human research ethics committee. The conduct and reporting of this project was guided by the Consolidated Criteria for Reporting Qualitative Research (CORE‐Q) checklist (see Appendix S1) (Tong et al., 2007).

### 2.2 Data collection

Sampling for this study occurred while participants were taking part in a national survey exploring alcohol use among Australian nurses and midwives. This survey was administered via the Qualtrics survey platform (Qualtrics, Provo, UT) and after completing questions on alcohol consumption, participants were asked whether they would like to take part in a telephone interview. The parent survey itself used a convenience sampling methodology, with invitations to participate distributed via nursing specialist organisations, unions, education providers and health departments. Participants who indicated they would like to take part in an interview were provided with the Participant Information Form and were required to provide an email address for a research assistant (DB, female) to contact them for an interview. Participants were reimbursed $50 for participation in the form of an electronic gift card, delivered via email. Of the 308 survey participants indicating their interest in a qualitative interview, 42 responded to email invitations to participate and completed the interview process (13.6%). We continued interviewing beyond theoretical data saturation to attain a wide range of nurses from diverse geographic and clinical settings.

The semi‐structured interview guide was developed following a scan of the literature and informed by our survey results (Searby et al., 2022) which explored alcohol consumption in the context of stress and the COVID‐19 pandemic. Statistical analysis of survey results showed that those who reported increasing alcohol consumption during the pandemic had higher consumption scores on a validated tool (the AUDIT), and we wished to probe responses to gather in‐depth information on how the pandemic had influenced nurse alcohol consumption. Demographic information, such as age, gender, years of experience and work setting, was collected to provide an overview of the workplaces and nursing experience of the participants, as shown in Table 1.

**TABLE 1:** Participant demographic characteristics

Characteristics	*n*	%	M	SD
**Gender**				
Female	41	98%		
Male	1	2%		
**Experience (years)**			24.17	13.15
**Role**				
Clinical Nurse	25	60%		
Clinical Nurse Consultant	1	2%		
Clinical Nurse Specialist	1	2%		
Education	3	7%		
Nurse Educator	1	2%		
Nurse Manager	8	19%		
Nurse Practitioner	1	2%		
Practice Nurse	2	5%		
**Work Setting**				
Alcohol and other drugs	6	14%		
Community health	1	2%		
Critical care	1	2%		
Diabetes	1	2%		
Diabetes education	1	2%		
Emergency department	1	2%		
General hospital	3	7%		
GP practice	2	5%		
Intellectual disability	2	5%		
Mental health	2	5%		
Mental health/alcohol and other drugs	2	5%		
Occupational health	3	7%		
Oncology	1	2%		
Ophthalmology	1	2%		
Palliative care	1	2%		
Perioperative	4	10%		
Policy	1	2%		
Remote health	1	2%		
Sexual health	1	2%		
Stomal therapy	2	5%		
Surgical	1	2%		
Tertiary institution	1	2%		
Vascular	1	2%		
Women's health	2	5%		
**State or Territory**				
Australian Capital Territory	2	5%		
New South Wales	10	24%		
Queensland	5	12%		
South Australia	2	5%		
Tasmania	1	2%		
Victoria	12	29%		
Western Australia	10	24%		

Interviews were conducted by the research assistant (DB, female) between July and August 2021. The interviewer has extensive experience in conducting qualitative interviews of this type. Interview duration ranged from 19:06 to 48:31 min, with a mean interview duration of 34:29 min (SD 7:14 min). A random sample of transcriptions was returned to participants to check for accuracy. We continued recruiting beyond theoretical data saturation to ensure that a wide geographical area was covered in our participant group. All interviews were audio recorded and transcribed by a professional transcription agency prior to data analysis, with both recordings and transcripts checked regularly by the lead author (AS, male) to ensure data quality. All transcripts were uploaded to the NVivo (QSR International, Version 20) computer program for analysis.

### 2.3 Analysis

Data analysis was performed by two members of the research team (AS & DB). After transcription, all data were uploaded to NVivo Version 20 for analysis (QSR International). We used a structural coding (Saldaña, 2013), a deductive approach where coding is aligned to the research question or aims, which in this instance were participant perceptions of COVID‐19‐related factors on their alcohol consumption. Coding followed the six‐step process outlined in Braun and Clarke's (2006) framework: familiarisation with the data, generating initial codes, searching for themes, reviewing themes, defining, and naming themes, and finally, producing the report. Both authors involved in the coding process completed the first two steps (familiarisation and generating initial codes) prior to reviewing initial themes together to ensure consistency in coding. We followed Hemmler et al.'s (2020) method of justifying coding decisions in order to reach consensus before progressing to searching and reviewing themes jointly. This method was selected to improve inter‐rater reliability, reduce bias and to accurately capture the perceptions of participants.

### 2.4 Findings

A total of 42 nurses took part in this study, 41 females and one male. Most participants were working in clinical nursing roles (*n* = 25, 59.5%) and held a mean number of years of clinical experience of 26.17 (SD 12.99). The highest representation came from the Australian state of Victoria (*n* = 12), followed by New South Wales (*n* = 10), Western Australia (*n* = 10), Queensland (*n* = 5), South Australia (*n* = 2), the Australian Capital Territory (*n* = 2) and Tasmania (*n* = 1). Once commencing the interview process, no participant withdrew their participation. Two parent themes emerged during data analysis: factors influencing alcohol consumption, and the pandemic's influence on alcohol consumption. Parent and subthemes are shown in Table 2.

**TABLE 2:** Themes and subthemes

Parent theme	Subtheme
Factors influencing alcohol consumption	Workplace factors External factors
2 The pandemic's influence on alcohol consumption	Increased consumption Moderation of consumption Alcohol as a reward

### 2.5 Factors influencing alcohol consumption

The first key theme in this paper reports factors that participants identified as influencing their alcohol consumption during the COVID‐19 pandemic. In the first subtheme, workplace factors that led to increased perceived stress and alcohol consumption were described. The second subtheme describes external factors that were attributed to increased alcohol consumption during the pandemic, particularly when lockdowns were in effect.

### 2.6 Workplace factors

As described in the background to this paper, the pandemic has had marked impact on nurse's perceptions of workplace stress. This stress was often related to the changed nature of nursing roles during the COVID‐19 pandemic, with some participants openly describing considering leaving their roles due to this stress. The following account demonstrates this point, describing fatigue and personal stress, and the ‘knock on’ effect of staff absenteeism and redeployment on nurses:

We've lost a lot of staff to the COVID screening and test and vaccines centres … I don't know if people aren't wanting to be in nursing at the moment. It is very challenging, and people are very tired. Staff are tired and that is reflected in a lot of personal leave, which makes it really hard for teams as well. (Participant 12)

A lack of resources, in this case personnel, was a common theme throughout the interview process. As a result, many participants described working overtime, including working on rostered days off, or where standard‐length shifts would have hours added due to insufficient nursing staff to maintain normal operations:

Even though I work full‐time, it is expected that you do overtime; you won't be replaced … so some days turn into 15‐hour days, at least once a week… I would say at least once a week one ten‐hour day turns into a 15‐hour day and usually either the eight or the ten [hour days] turn into a twelve‐hour day. (Participant 28)

Even outside settings associated with increases in presentations, such as emergency departments and hospital respiratory settings, participants described strong pressure to do overtime or longer shifts, as in the following example from a maternity setting:

… since COVID the birth rates seem to have gone up exponentially. And we have to accommodate them 24/7 and it causes great stress because often we don't have a theatre or staff available … So that again cases pressure on us doing overtime. We do a lot of overtime just to accommodate all the emergency cases for maternity. (Participant 13)

Increasing shift length was described by participants as a strategy to cope with increasing workloads and the need to cover absent staff, particularly in acute hospital settings. Although this strategy may have provided cover for staff shortfalls, participants described increasing their own absenteeism because of exhaustion or burnout:

In the last few weeks … it's been getting quite bad, and the management are trying to make different solutions, because we've had to do a lot of double shifts, from afternoons to overnight … I'm not wanting to go to work as much, and I'm wanting to take more sick days, but then I feel guilty for taking more sick days because I know that I won't be replaced. And I know that there's other nurses on my ward who have just taken two weeks’ off because they just can't do it anymore, they're too stressed and not coping. (Participant 17)

Not only did increasing shift length and forced overtime result in exhaustion and burnout, but as the following participant describes, resentment at being ‘stuck’ in the workplace and missing activities outside work:

I've missed, you know this and that, and I've felt that there was some correlation with overtime and being stuck there and knowing that, you know … resentment when it got to that. (Participant 33)

Workplace stressors such as those described above were frequently linked to increased alcohol consumption during the interview process. As seen in the following example, workplace anxiety due to the unpredictable nature of nursing work was linked to increased alcohol consumption:

COVID has created a very unsure working environment, it's the lack of communication, the mixed communication, the not knowing what's going to happen next. We used to be able to come to work and almost predict that every day we'd come to work, now we've got to look and see are we working today, are we not. So, all of that unstable work environment is causing anxiety and people are drinking more. (Participant 30)

When queried as to whether stressful work conditions, long shifts and overtime had an impact on alcohol consumption, participants often responded that drinking alcohol was a means to reduce the stress experienced in the workplace:

… I was looking at that [alcohol consumption] as a way to destress and to relax, particularly by the end of the week. I did find that I was going and purchasing a bottle of wine, which normally I wouldn't even have wine in the house … Normally, I would never really think to go and get alcohol unless I was specifically going to an event. (Participant 25)

In addition, participants related their increased alcohol consumption to a perception of increasing work stress. The following account describes triggers occurring in the workplace:

When the stress started … I was going through all this crap at work and I just thought ‘oh I just need a drink’, and searching for alcohol … I was searching and I would have done anything … It was, it was terrible but then I bought alcohol online, and pretty much started drinking most nights … I would be good for a little while then, you know something would be exacerbated at work … I did find the lockdown stressful, and it would escalate again. (Participant 33)

### 2.7 External factors

In addition to workplace factors that influenced alcohol consumption, participants also described several external factors occurring during the pandemic and related lockdowns that had an impact on their drinking behaviour. Many of these factors related to lockdowns that were introduced to halt SARS‐CoV‐2 transmission combined with perceived work stress:

I have a lot of other things going on and yeah, I kind of got into a pattern of just drinking more, just because I had my dad living here and it's just sad because that's what he did. So, I don't think it was just work, but yeah, I think sometimes you get home from work and think ‘I really deserve a drink’. (Participant 10)

As reported elsewhere, lockdowns have been a significant source of personal stress in regions where they have frequently occurred. As the following participant noted, and as reflected in the general population, lockdowns often led to the purchase of alcohol:

We went into lockdown the other week, and my first thought was, ‘Oh, better get a wine’. [Laughs] It wasn't like, I try not to drink on school nights, but that would have been a Thursday, Wednesday, or something we were going into lockdown, and yeah, I bought a bottle of wine. (Participant 16)

During lockdown, the removal of opportunities to participate in usual activities was also cited as a driver of increased alcohol consumption. For example, the following participant reported increased consumption due to an inability to participate in exercise:

I admit it [alcohol consumption] did go up when I was not able to exercise. (Participant 18)

In addition to a reduction in usual activities, remote learning was also used to reduce physical contact in schools. Many participants described increased stress due to a ‘dual role’; working as a nurse in clinical settings, and supervising the learning of their children around paid employment:

… I noticed my alcohol consumption actually increased during that time because I was home schooling. So instead of having drinks on just the set Friday, Saturday night that I normally do, just one drink, I was finding myself after a very hard day of home schooling that the kids were in bed and I would have a glass of wine, maybe three times a week. (Participant 4)

Further, ‘work from home’ orders were described as both stressful and leading to increased alcohol consumption. As the following account indicates, working from home was not only a changed work practice, but had implications in large households:

Currently it's tricky because I've been working at home for 18 months now which is bizarre because to me in the 39 and a half years that I've been working, I've never worked from home ever… working from home from a small apartment with four adults so it's been quite challenging. (Participant 9)

These accounts indicate that not only work stress played a significant role in increasing alcohol consumption during the pandemic, but consistent with reports from the general population, external factors such as lockdowns, remote learning and working from home were also described as having a tangible impact on alcohol consumption.

### 2.8 The pandemic's influence on alcohol consumption

In the second key theme, participants described the influence of the pandemic on their own alcohol consumption. Mostly, participants who reported consuming alcohol prior to the pandemic described changes in their level of alcohol consumption, commonly increasing the number of weekly days of alcohol consumption, and a realisation that their consumption increased and needed to be moderated. Of those participants who reported minimal or no alcohol consumption prior to the pandemic, little change was described. Finally, several participants spoke of alcohol as a ‘reward’ for working in stressful conditions encountered during the pandemic.

### 2.9 Increased consumption

In the early stages of the pandemic in Australia, a reduction in hospital presentations was noted. Participants who worked in settings where presentations were reduced or resources were redirected to areas where early SARS‐CoV‐2 infections were hospitalised were often ‘stood down’, having their hours reduced due to the decreased workload. Some participants who were in this situation described an increase in their alcohol consumption in response, which then continued once they returned to work, and as their workload increased:

At the start we were so quiet because people stopped presenting to hospital. [Work was] dead and everyone was getting time off, so we were drinking a bit more. And then when we started getting slammed, because they'd put a pause on all the elective surgery, so when all they started coming in as emergencies was when it picked up as well. And then you have a really bad shift and then you come home and just take the edge off. (Participant 39)

In areas where lockdowns did not occur as frequently, staff who had their hours reduced described increasing social events where alcohol consumption occurred:

All the perioperative staff were informed, it didn't matter if you were part‐time, full‐time, you'll be doing six hour shifts instead of ten‐hour shifts. That wasn't per day, so it was a twelve‐hour week that you'll be doing. So, everyone's hours significantly decreased. I think it was for maybe about three to four weeks. And you could have taken annual leave or your long service leave. Most of us … I would say maybe about 35% of the workforce in theatre took the twelve hours a week, whereas everyone else took annual leave and long service leave. And during that time there wasn't a lot to do so there were little groups that got together, we'd have barbecues or whatever, lunch or dinner, and drinking was involved in those times. (Participant 28)

Alternative technologies such as Zoom were often described as ways to maintain social contact where social gatherings were prohibited. However, as one participant described, Zoom gatherings frequently involved alcohol consumption:

I'm lucky if I have a couple of glasses a week you know. And I think that was starting to maybe creep up a little bit because that was a way of connecting and so people would sort of sit on the Zoom calls, you know on a Friday late afternoon or something, you know let's have a drink. (Participant 9)

Although depictions of increasing alcohol consumption expressed often related to the pandemic, some participants indicated that the stress experienced in the nursing profession predated the COVID‐19 pandemic:

[It] definitely wasn't discussed that as a nurse you will definitely be exposed to a high level of stress in almost any job that you have and you should really keep an eye on your alcohol consumption, not that I would say that I didn't actually consume more than recommended before I did my nursing degree. (Participant 2)

While participants who described increased alcohol consumption in the context of the pandemic did readily describe constraints around their drinking, such as needing to work without being under the influence of alcohol, there were accounts where colleagues had reported for duty intoxicated:

One of them [staff] has unfortunately taken it too far and had to be sacked because he came in drunk. He drove in and then drank himself in a parking area and got to the handover very inebriated. (Participant 38)

Although the above example was an outlier, it does indicate that there were healthcare staff whose consumption of alcohol resulted in the termination of their employment. However, none of the participants in this study described their losing their personal employment due to alcohol consumption. When queried, they were also able to describe where to seek help if needed, with most participants able to describe their local Employee Assistance Program (EAP) as a first point of contact if help was needed with alcohol consumption.

### 2.10 Moderation of consumption

Several participants reported moderation of their alcohol consumption, often in response to the realisation that their drinking behaviours were beyond accepted norms. Often, these efforts involved reducing alcohol consumption, such as not drinking on weekdays:

It was just a nice thing to have a glass of wine and then when you stop and think at night, you're having a glass or two of wine and it just crept up on you. And then I made the decision I didn't want to do that. So, for a while, not that I struggled with it, like missed it, but now I specifically try not to drink during the week at all. (Participant 10)

Other participants reported that the constraints of their work provided a degree of moderation of their alcohol consumption, with the following participant describing the need to be ready for work (on call) as a means of moderating consumption:

Luckily work often doesn't allow me to drink because we do a lot of on‐call. But otherwise yes, I would drink each night. (Participant 13)

Participants who disclosed that they had previously ceased consuming alcohol reported that their cessation of drinking had been beneficial during the pandemic; the following participant spoke openly about the perceived impact that alcohol would have on their present resilience if they had still been drinking when the pandemic arrived:

I quit drinking a year before the pandemic and I've been able to reflect that thank goodness I stopped drinking before the pandemic because if I was drinking now, I would not be coping as well as I am now. So, I think that has helped me be on the straight and narrow. (Participant 1)

As the following account indicates, there was a strong view that consuming alcohol alone was problematic, especially in stressful times such as during the COVID‐19 pandemic, with this view being a contributing factor to moderating consumption:

It's shunned, it's not seen as, like to go out and drink with people is social, it is acceptable, but to say you, ‘I went home and I drank half a bottle of wine’, people would go, ‘What's her problem?’ or, ‘What's his problem?’ or, ‘Why are you doing that?’ I think people would be questioned. I think people do feel guilty about it because they know potentially it's probably not the right thing to do. They should probably be doing, destressing other ways, gym, or running, or engaging with friends. (Participant 16)

Despite several reports of increased consumption, many participants described efforts to moderate their consumption. This finding indicates that participants had good insight into their alcohol consumption during the pandemic and were able to make efforts to reduce their consumption in recognition that either the quantity of alcohol or frequency of drinking occasions were increasing.

### 2.11 Alcohol as a reward

Analysis of the data revealed a strong theme of participants viewing alcohol consumption as a reward for working under conditions where resources were stretched, the workload heightened, or additional hours were worked due to staff absence. Mostly, participants who described an increase in their drinking behaviour attributed this to a reward for these demanding work conditions. As the following account reports, this was a common theme among peers:

I'm longing for a glass of wine when I come home or already on my last hour at work. And that seems to be the topic of the day as well; I can't wait to open my bottle of wine. (Participant 13)

As the following account indicates, the consumption of alcohol was considered ‘compensation’ for arduous conditions and working through situations such as missing scheduled breaks:

I didn't have lunch, didn't have time, you know, what are you taking for lunch, oh I'm not I don't have time to eat it. What do you mean, you've got to have a lunch break, you know? I think resentment made me think oh I've got to have a drink, yeah … so there's the reward. I'll pat myself on the back … nearly everyone I've spoken with from nursing to any other profession have been drinking more during COVID … it's incredible, yeah. (Participant 33)

Further, the following participant spoke of alcohol consumption as not only a reward, but a mechanism to cope with workplace stress in the absence of other means to cope, expressing a belief that heavy consumption of alcohol often went hand in hand with a bad day:

I think when they've had a really rugged day they often go home and … if they haven't got other things in their life, they often reach for what's closest and that's probably a nice glass of wine and that obviously starts off with one then leads to two and then next comes the bottle I think. I hear that from a lot of people. (Participant 37)

As the following participant described, the ritual of rewarding oneself with alcohol often went beyond work stresses, becoming habitual:

A lot of times when I came home from work I was like, ‘Yes, now I can have a drink’. It's 5 o'clock, yep, I can have a drink. But I have to say I can also do that when I'm not stressed. I was doing that when I wasn't stressed, but I was just really looking forward to having a drink. (Participant 27)

Participants also recognised that among peers, using alcohol both as a reward and a means to cope with workplace stress was openly discussed:

It's a different coping mechanism … the ‘Bring alcohol’ adds a little bit of a joke and light‐heartedness about what the situation is. (Participant 25)

The perceptions of alcohol consumption expressed by participants during the semi‐structured interview process indicate that although consuming alcohol alone was frowned upon during the pandemic, there were several opinions and observations that showed alcohol was commonly described as an acceptable means to mitigate the stress experienced during the pandemic. Participants often spoke of alcohol as a reward for working overtime, missing breaks and working on the ‘front line’ when most of their friends and family had transitioned to working from home.

## 3 DISCUSSION

Our findings indicate that alcohol consumption during the COVID‐19 pandemic increased due to several stressors, including those encountered in the workplace. Notably, many participants described increasing alcohol consumption in response to several workplace factors, including overtime, missed breaks and reduced staffing and resources. Conversely, reduced hours in response to the shut down or reduction of clinical areas were also described as drivers of change to alcohol consumption. These findings mirror those found across the general population, particularly during lockdowns (Mongeau‐Pérusse et al., 2021; Sallie et al., 2020). Alcohol consumption has been shown to have a direct impact on personal well‐being (Parackal & Parackal, 2016), with heavy alcohol use shown to increase the susceptibility to burnout and absenteeism (Ahola et al., 2006; Cunradi et al., 2005; Fagin et al., 1996; Pedersen et al., 2016). Although being a nurse itself was often described as stressful, pressures related to COVID‐19 created an extra burden on participants, many of whom were reconsidering their place in the nursing workforce.

Staffing issues among the nursing workforce were often described in our study as being problematic, particularly where the solution to these issues was to enforce longer shifts or expect nurses to work double shifts (overtime) to cover staff shortages. As our data indicate, this measure to address staffing shortfalls often created more issues, with nurses forced to work overtime taking sick leave due to exhaustion or burnout. Although there is evidence that longer shift lengths and shorter roster pattern rotations reduce SARS‐CoV‐2 infections among healthcare workers (Kluger et al., 2020), studies support our finding that longer shift length is attributed to poor well‐being and mental health during the pandemic (Shaukat et al., 2020). Research exploring 12‐hour nursing shifts prior to the onset of the pandemic found that fatigue had an impact on patient safety and an increased intention to leave their profession (Dall'Ora et al., 2022), further highlighting that enforced overtime to cover staffing shortages may actually endanger both the nursing workforce and patients they care for.

In addition to the workplace factors described by participants in this study, there were also several factors external to the workplace that contributed to increased alcohol consumption. These factors included lockdown and ‘work from home’ orders and spillover effects from these measures, such as remote learning and overcrowding in accommodation. For those working from home, conflicts between family and work have been shown to increase perceived stress (Galanti et al., 2021). Although not arising in our semi‐structured interview process, a previous study of healthcare workers also found significant stress relating to the fear of contracting the SARS‐CoV‐2 virus and transmitting it to family members (Sheen et al., 2022).

Increased alcohol use has also been associated with thoughts of suicide or self‐harm during the pandemic. Bismark et al. (2022) analysed data from a survey of frontline healthcare workers (*N* = 7795), finding those who reported thoughts of suicide and self‐harm (*n* = 819) reported increased alcohol consumption (OR 1.58). Adverse mental health was also associated with increased alcohol consumption in a cohort of Australian doctors (*n* = 1966) surveyed in 2020 (Pascoe et al., 2021), and among 3678 German healthcare workers with symptoms of depression and anxiety (Morawa et al., 2021), indicating that alcohol is both associated with reports of mental ill health in healthcare workers. Given previous research has indicated that healthcare workers often report mental ill health due to anxiety and workforce pressures during the pandemic (Holton et al., 2021; Saragih et al., 2021), further investigation into the relationship between mental ill health and alcohol consumption in healthcare workers is urgently required.

### 3.1 The long‐term impact of alcohol consumption among nurses

While the long‐term impact of the COVID‐19 pandemic to nursing is mostly believed to be due to attrition, burnout and self‐reported symptoms of mental ill health (Holton et al., 2021; Huerta‐González et al., 2021; Pérez‐Raya et al., 2021; Squires et al., 2022), our research indicates that alcohol consumption must be included in this conversation. Although several participants spoke of measures to mitigate their alcohol consumption during the acute stages of the COVID‐19 pandemic, the healthcare response to managing and treating the SARS‐CoV‐2 virus has extended beyond what many thought would occur in 2020, particularly with the widespread uptake of vaccines. However, case numbers, hospitalisations and death rates remain high globally, indicating a high burden to healthcare services and workers (World Health Organization, 2022).

To complicate the issue of a ‘long tail’ of COVID‐19 for healthcare services and workers, many jurisdictions are reporting a shortage of nursing staff; this shortage is driven by both an inability to find nurses and reports of high intention to leave the profession among existing staff (Cornish et al., 2021; Schug et al., 2022). As nurses are at the forefront of caring for patients with the SARS‐CoV‐2 virus in both hospital and community care, it is reasonable to assume that stress related to workforce shortages as described in our study (lack of staff, excess overtime, lack of resources) will continue for the foreseeable future. As our findings have shown, several nurses are resorting to increased consumption of alcohol to manage this stress.

An ethnographic study of 21 hospital nurses in the United States in 2001 found no difference in alcohol consumption between nurses describing working in high‐stress situations and those not; however, several protective factors were identified that may not be attainable or present during the COVID‐19 pandemic (Moore, 2001). These factors include ‘talented and caring co‐workers’, ‘independent work’ and ‘professional growth’. As previous literature and our findings indicate, the pandemic has resulted in the reduction of nursing resources and a need to abandon all other priorities to provide safe care for a large cohort of SARS‐CoV‐2 patients (Akkuş et al., 2022).

As discussed, research indicates a strong association between mental ill health and increased alcohol consumption. Over the long term, increased alcohol consumption has been shown to contribute to burnout, intention to leave and absenteeism (Pidd et al., 2006; Roche et al., 2016). As established in this paper, the current nursing workforce reports high levels of perceived stress, and often intention to leave, and increasing alcohol consumption adds to this complex issue and poses a substantial threat to healthcare systems that are strained. Additionally, issues associated with increasing alcohol consumption pose a threat to nursing workforce sustainability (Drennan & Ross, 2019). Globally, recruitment and retention of nurses were substantial issues prior to the pandemic, and efforts should be made to prevent exacerbation of preventable workforce sustainability issues where possible (Oulton, 2006; Shaffer et al., 2022). There are also significant mental and physical effects of long‐term alcohol consumption beyond recommended guidelines (Conigrave et al., 2021). The COVID‐19 pandemic has indicated that a significant investment in nursing workforce well‐being is sorely needed to retain a key component of the healthcare workforce that is pivotal in responding to public health crises such as COVID‐19.

### 3.2 Strengths and weaknesses

To our knowledge, our study is the first exploring the qualitative perspectives of alcohol consumption among nurses during the COVID‐19 pandemic. However, there are limitations that need to be considered when interpreting these data. Our sampling methodology involved recruiting nurses through professional organisations; therefore, our sample may reflect older, more experienced nurses who are engaged with their professional association rather than a broad spectrum of all nurses (e.g. neophyte nurses and graduates). Further, the sampling methodology resulted in only one male included in the interview process. Our research is cross‐sectional in nature and represents the subjective experience of the participants; we recommend repeating this research once the pandemic is declared over to determine whether the alcohol consumption behaviours described by participants continue.

## 4 CONCLUSION

The COVID‐19 pandemic has resulted in significant stress to healthcare services and workers, and nurses are in no way immune. Increased stress has resulted in increasing alcohol consumption among some nurses and has been openly described to cope or ‘reward’ oneself for working in a situation where resources and personnel were often scant. Increased alcohol consumption has substantial long‐term implications for nursing staff both at a personal and workforce level, and research shows it to be strongly associated with the mental ill health experienced by nursing staff during the COVID‐19 pandemic.

## 5 RELEVANCE TO CLINICAL PRACTICE

Quality clinical care requires a workforce that is responsive and adaptable; COVID‐19 has made this difficult due to stress experienced by nurses. Our findings indicate that our participants described increased alcohol consumption in response to this stress. Additionally, external stress experienced in the form of lockdowns, home schooling and other complexities of social distancing related to the COVID‐19 pandemic were described as having a compounding effect on stress levels. As research indicates, increased alcohol consumption often leads to increased absenteeism (Roche et al., 2016) and has been associated with burnout in health professionals (Galaiya et al., 2020; O'Kelly et al., 2016). Both factors have been implicated in ‘intention to leave’ the nursing workforce, potentially leading to a shortage of experienced nurses (Moloney et al., 2018).

Longer shift lengths have been studied in nursing, finding that fatigue leads to negative effects on safe, quality care (Dall'Ora et al., 2022). Although not specifically researched in the context of the COVID‐19 pandemic, where nurses often remained ‘on shift’ due to staff furloughing and shortages, research has highlighted extended working hours as detrimental to quality care and nurse well‐being (Nymark et al., 2022; Yayla & Eskici İlgin, 2021). On a personal level, our participants described shift length as a key driver of their increased alcohol consumption. These stressors, combined with increased alcohol consumption, may have a deleterious effect on safe and quality care.

## AUTHOR CONTRIBUTION

Each author certifies that their contribution to this work meets the standards of the International Committee of Medical Journal Editors.

## CONFLICT OF INTEREST

None to declare. No external funding was received by any authors for this study.

## PATIENT OR PUBLIC CONTRIBUTION

No patient or public contribution: our work explores alcohol consumption among nurses.

## Supporting information


Appendix S1

## Data Availability

The data that support the findings of this study are available on request from the corresponding author. The data are not publicly available due to privacy or ethical restrictions.
